# Designing 3D Membrane Modules for Gas Separation Based on Hollow Fibers from Poly(4-methyl-1-pentene)

**DOI:** 10.3390/membranes12010036

**Published:** 2021-12-27

**Authors:** Svetlana Yu. Markova, Anton V. Dukhov, Martin Pelzer, Maxim G. Shalygin, Thomas Vad, Thomas Gries, Vladimir V. Teplyakov

**Affiliations:** 1 A.V. Topchiev Institute of Petrochemical Synthesis RAS, 29 Leninskiy Prospect, 119991 Moscow, Russia; anton.duhov.97@mail.ru (A.V.D.); mshalygin@ips.ac.ru (M.G.S.); tepl@ips.ac.ru (V.V.T.); 2 Institut für Textiltechnik of RWTH Aachen University, Otto-Blumenthal-Straße 1, 52074 Aachen, Germany; Martin.Pelzer@ita.rwth-aachen.de (M.P.); Thomas.Vad@ita.rwth-aachen.de (T.V.); Thomas.Gries@ita.rwth-aachen.de (T.G.)

**Keywords:** gas separation membrane, poly(4-methyl-1-pentene), hollow fibers, 3D braided hollow fiber membrane structures

## Abstract

Designing hollow fiber (HF) membrane modules occupies one of the key positions in the development of efficient membrane processes for various purposes. In developing HF membrane modules, it is very important to have a uniform HF distribution and flow mixing in the shell side to significantly improve mass transfer and efficiency. This work suggests the application of different textile 3D HF structures (braided hoses and woven tape fabrics). The 3D structures consist of melt-spun, dense HFs based on poly(4-methyl-1-pentene) (PMP). Since the textile processing of HFs can damage the wall of the fiber or close the fiber bore, the membrane properties of the obtained structures are tested with a CO_2_/CH_4_ mixture in the temperature range of 0 to 40 °C. It is shown that HFs within the textile structure keep the same transport and separation characteristics compared to initial HFs. The mechanical properties of the PMP-based HFs allow their use in typical textile processes for the production of various membrane structures, even at a larger scale. PMP-based membranes can find application in separation processes, where other polymeric membranes are not stable. For example, they can be used for the separation of hydrocarbons or gas mixtures with volatile organic compounds.

## 1. Introduction

The development and practical application of membrane processes have stimulated active research on membrane materials and methods for forming membranes and designing membrane modules. Over the past several years, the interest in hollow fiber (HF) membrane modules has constantly increased. Their main advantages include the following: they have high packing densities and specific productivity, and wide areas of application both in gaseous and liquid media [1]. Advances in membrane material science, the features of selective gas transfer in polymer membranes and the modeling of membrane processes and foundation are systematically and thoroughly presented in the literature [2,3,4,5,6]. The HFs proposed by J. Hennis and M. Tripody [7] focus on gas separation. Nowadays, HFs are used for liquid purification, including pervaporation, membrane distillation, various types of osmosis, gas/liquid and liquid/liquid membrane contactors. Ecologically non-friendly phase inversion methods are mainly used for the production of polymer HFs [4,7,8] and flat-sheet membranes [9,10]. Recently, increasing attention has been paid to the environmental problems connected with such production, such as the disposal of solvents and the residue of solvents in the fibers after production. Therefore, production processes for HFs using environmentally friendly methods are being developed. The latest achievements in the production of polymeric HF membranes from an environmental and health point of view include the melt/solution integrated homogeneous reinforcement method, the melt spinning–stretching interfacial phase separation method or the utilization of “green” solvents [11].

Unlike most polymer membranes produced with organic solvents, membranes based on poly(4-methyl-1-pentene) (PMP) can be obtained by the melt spinning method without using organic solvents [12]. Thermoplastic PMP is a semicrystalline aliphatic polyolefin, with a glass transition temperature T_g_ of 20–30 °C and a melting temperature T_m_ of 230 °C. It has the lowest density compared to all other thermoplastic materials (0.83 g/cm^3^) [13]. PMP has high gas permeability coefficients compared to commercial membrane polymers such as polysulfones, polycarbonates, polyimides and their functional derivatives. Other important advantages of PMP include the following: it can be used in industrial production, can form membranes by melt spinning technology, is stable in the presence of C_3+_ hydrocarbons and vapors of organic solvents and has high chemical resistance [14]. These advantages allow it to be considered a promising material for the production of membranes, particularly for gas separation processes, regardless of the moderate level of selectivity. For example, PMP-based membranes have been investigated for gas separation in several studies [15,16,17,18].

A technological process used for preparing PMP-based “Graviton” HFs was developed in the 1980s [19]. Unfortunately, in applying membrane modules based on “Graviton” HFs in real gas separation processes, it was discovered that the HFs were blocked due to the capillary condensation of water vapors inside the fiber as a result of its small internal diameter. Today, membranes based on PMP are mainly used for extracorporeal membrane oxygenation (“artificial lungs”) [20,21,22]. PMP has high resistance towards organic components and thus is promising for the separation of hydrocarbons and complex gaseous media with volatile organic compounds (VOCs).

While the formation of various HFs using conventional production processes can be considered sufficiently advanced, membrane modules based on HFs are being actively improved. Table 1 presents the main trends in improving the design of HF membrane modules.

Table 1 shows that the design of HF membrane modules occupies one of the key positions in the development of efficient membrane processes for various purposes. Improvements are aimed at increasing the efficiency of gas separation, membrane oxygenation, gas/liquid and liquid/liquid membrane contactors, FO, membrane distillation and filtration, energy recovery during the desalination of seawater by FO and PRO and modeling membrane processes. In fact, the actual results of the fundamental research in chemical engineering have led to a wide range of practical applications. This work focuses on the development of new membrane modules based on 3D structured HFs obtained by reagent-free technologies [12]. In particular, 3D membrane structures based on PMP HFs are suggested to improve the characteristics of HF membrane modules by increasing mass transfer on the shell side due to the regular distribution of HFs and creation of flow mixing, and avoiding the problem of channeling. PMP HF 3D structures are realized by textile processes—in particular, triaxial braiding and tape weaving. For the triaxial braiding, the number of braiding threads and the braiding angle are varied. For the tape-woven fabrics, different weft densities and binding methods are used. This leads to the realization of different membrane module geometries, which opens up new possibilities for the improvement of membrane processes. The application of textile processing can be harmful to HFs. Damage of HFs’ walls or their collapse will lead to the loss of their membrane properties. The aim of this work is to investigate the gas transfer and separation characteristics of the newly developed 3D membrane structures based on PMP HFs compared to the same standalone PMP HFs in order to check their integrity and ensure that the HFs do not collapse after weaving and braiding processing.

## 2. Materials and Methods

### 2.1. Hollow Fiber Preparation

**Figure 1:**
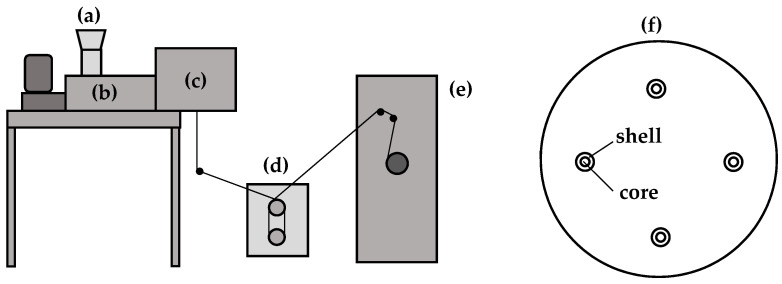
Laboratory-scale melt spinning setup and sketch of the 4-hole spin plate: (**a**) hopper; (**b**) extruder; (**c**) spin head; (**d**) godet duo; (**e**) winder; (**f**) 4-hole core–shell spin plate with supporting air in the core.

**Figure 2:**
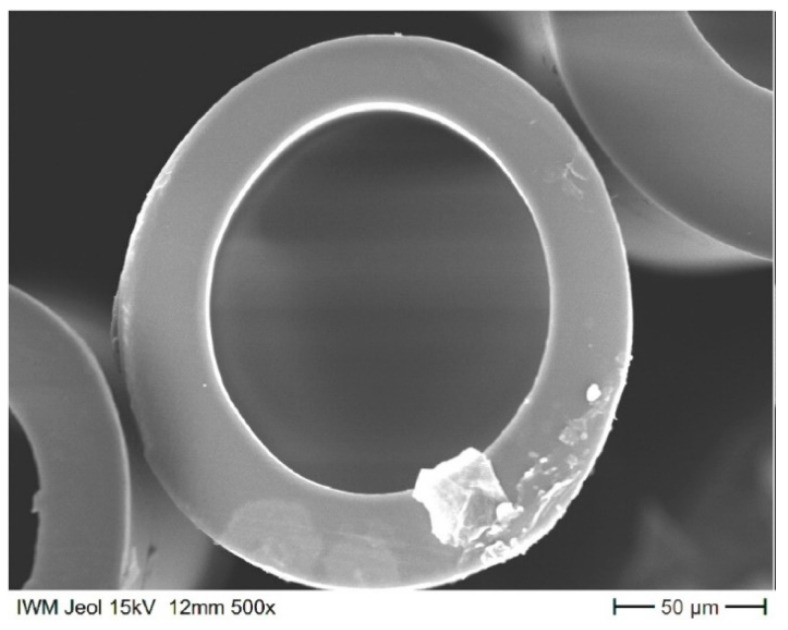
HF cross-sections.

PMP type MX002, obtained from Mitsui Chemicals, Inc. (Minato, Tokyo, Japan), was used to prepare non-porous HFs with dense walls. PMP HFs were produced by the melt spinning process with a four-capillary core–shell spinneret and supporting air in the core using a laboratory-scale plant from Fourné Polymertechnik GmbH (Alfter, Germany). The preparation details of the HFs are given in [12]. Air was introduced in order to prevent the fibers from collapsing after extrusion, when the melt strength beyond the capillaries is not yet strong enough. The spinning process setup and a sketch of the 4-hole spin plate are depicted in Figure 1. The internal diameter of the HFs was around 3 times larger than that of the industrial “Graviton” HFs. This can help to prevent the problem of vapors’ capillary condensation and blocking of HFs. A cross-section of a PMP HF obtained is shown in Figure 2. The HF almost has a round geometry, with slight deviations and non-uniformity of wall thickness. The geometry of the HFs was characterized by measuring the external and internal diameters of several HF samples in two perpendicular directions and averaging their diameters and wall thickness values. The average external and internal diameters were found to be equal to 187 and 111 µm, respectively; the average wall thickness was found to be equal to 38 µm. The processing parameters used for the preparation of the PMP HFs are presented in Table 2. The T_g_ of the obtained HFs was found to be 21 °C.

### 2.2. 3D Braided and Woven Tape Fabrics

**Figure 3:**
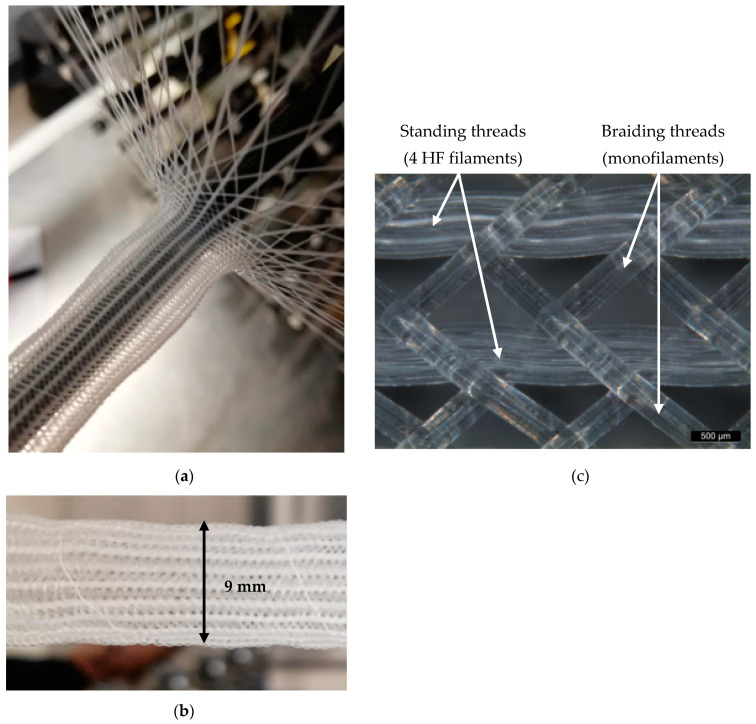
(**a**) Preparation of 3D structured HF fabrics via triaxial braiding; (**b**) braided hose; (**c**) structure of braided HF hose with medium braiding angle.

At the Institut für Textiltechnik (ITA) of RWTH Aachen University, Germany, 3D structured fabrics were developed based on the HF described in Figure 3a,b. Different triaxial braided hoses were produced based on different braiding angles and numbers of standing threads. PMP monofilaments (not hollow) of the same fineness were used as braiding threads. Either 24 or 48 braiding threads were used in order to make the textile less or more dense, respectively. In each case, the number of standing threads parallel in the axial hose direction was 24 (each with four HFs). In Figure 3c, the tubular fabric with 48 braiding threads is depicted. In this work, braided HFs with a medium braiding angle (45°) were investigated.

**Figure 4:**
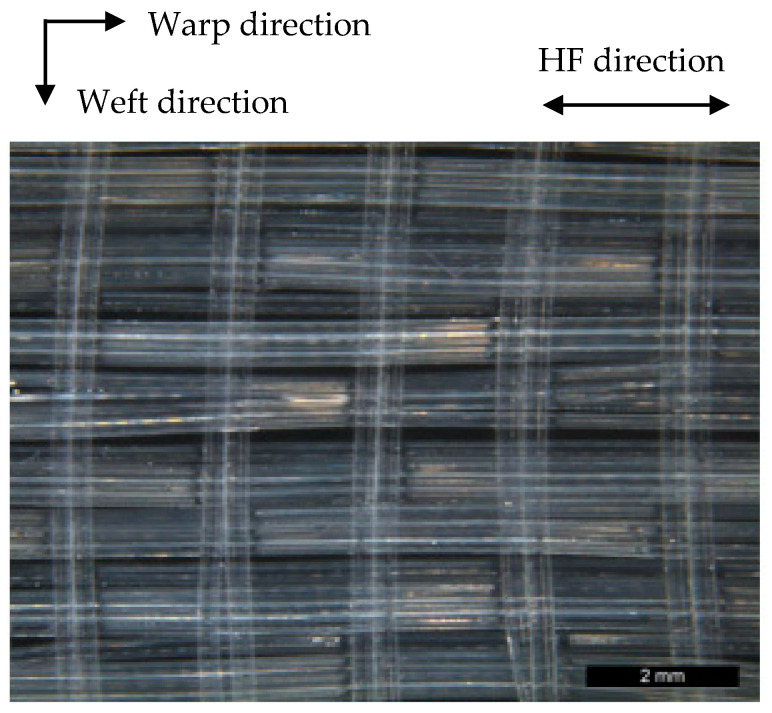
Woven HF fabric.

The woven tape fabrics were produced using different bindings: twill binding and atlas binding. In the woven fabrics, 24 HF threads (each with 4 HF filaments) were used in the warp direction and monofilaments in the weft direction (Figure 4). The warp density was varied from 5/cm to 15/cm. In fact, the preparation of 3D braided HF membrane structures can use the advantages of textile techniques, including variation of all parameters. Nevertheless, it is extremely important to keep HFs from collapsing, especially in the crossing points of warp and weft yarn. Moreover, special attention should be paid to gentle fiber handling when textile processing the HFs in order to prevent damage of the dense walls.

### 2.3. Gas Permeability Measurements

**Figure 5:**
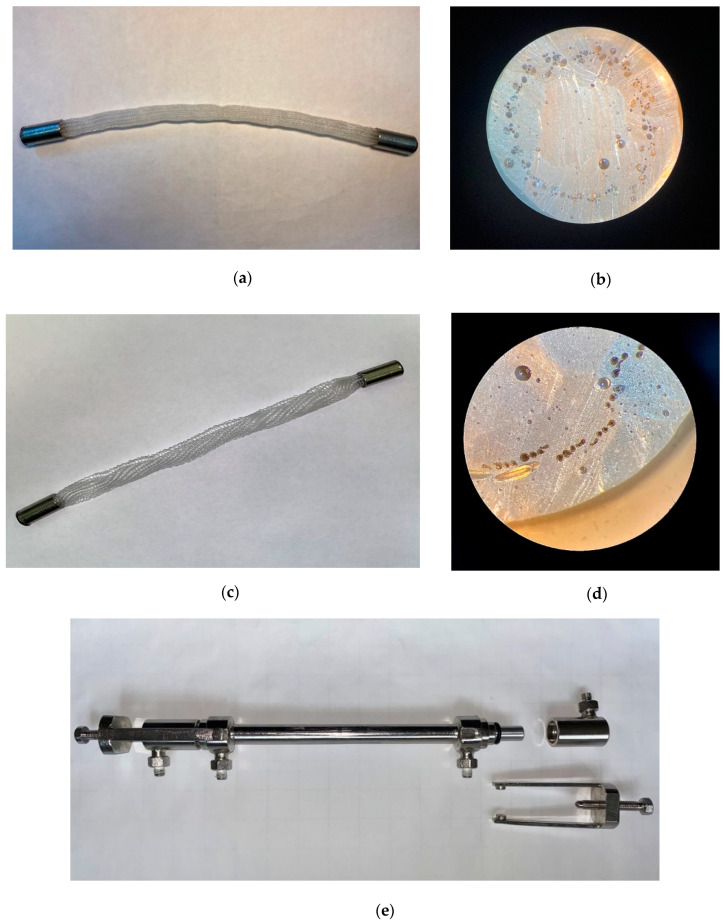
Lab-scale membrane module: (**a**) sealed 3D triaxial braided hose; (**b**) cross-section of the bore-side sealed entrance part; (**c**) sealed woven fabric (rolled); (**d**) cross-section of the bore-side sealed entrance part; (**e**) complete assembly of lab-scale membrane HF module.

To measure the permeability of the 3D braided and tape-woven fabrics, laboratory membrane modules in metal design were created at the A.V. Topchiev Institute of Petrochemical Synthesis RAS (TIPS RAS), Moscow, Russia. The ends of the fabrics were placed into metal sleeves and encapsulated by a constructional adhesive based on epoxy resin (Figure 5a,c). Protruding ends of HFs were cut when the adhesive was completely cured (Figure 5b,d) to ensure that all HF were opened. An overall view of the metal module is shown in Figure 5e. The working area of the HFs in the module was 70 cm^2^.

In order to test the integrity of the HFs and to preserve their gas transport and separation characteristics after the formation of 3D structures, gas permeability was measured with a CO_2_/CH_4_ mixture (50/50 vol.%), which was used previously to test initial PMP HFs [40].

The permeability measurements of laboratory membrane modules were carried out in the temperature range of 0 to 40 °C on a differential type set-up with gas chromatographic analysis. A detailed description of the experimental measurements of HFs can be found in [40].

The permeability coefficient through the HFs was calculated using the equation:

(1) $$P_{i}=\frac{J\cdot c_{i}^{\prime }\cdot L}{A\cdot p\cdot \left(c_{i}-c_{i}^{\prime }\right)},$$

where *P* is the permeability coefficient, Barrer (7.5 × 10^−18^ (m^3^⸳m)/(s⸳m^2^⸳Pa)); *J* is the flow rate of the gas carrier with the gas studied that exits from the bore side, m^3^(STP)/s; *L* is the HF wall thickness, m; *A* is the working membrane area, m^2^; *p* is the atmospheric pressure, Pa; *c_i_′* is the concentration of the gas studied in the gas carrier stream from the bore side, vol.%; *c_i_* is the concentration of the gas studied in the shell side, vol.%.

The selectivity of the gas pair was calculated as follows:

(2) $$\alpha_{ij}=\frac{P_{i}}{P_{j}}$$

The following equation was used for the approximation of the temperature dependence of the permeability coefficient:

(3) $$P=P_{0}\exp\left(-\frac{E_{P}}{RT}\right),$$

where *E_p_* is the apparent activation energy of permeability, J/mol; *T* is the temperature, K.

## 3. Results

Gas permeability coefficients for CO_2_ and CH_4_ obtained for triaxial braided hose and woven fabric with PMP HFs are presented in Table 3.

**Figure 6:**
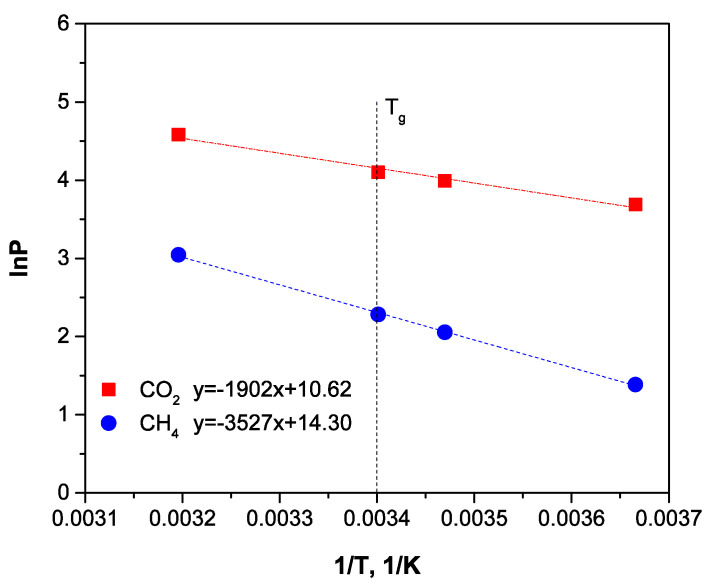
Temperature-dependent permeability coefficient of the triaxial braided hose membrane module for CO_2_ and CH_4_ in the mixture (50/50%vol.).

**Figure 7:**
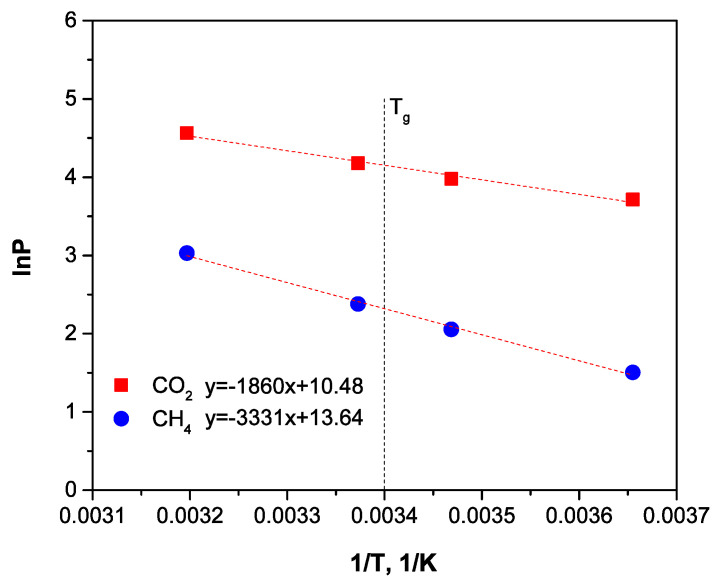
Temperature-dependent permeability coefficient of the tape-woven fabric membrane module for CO_2_ and CH_4_ in the mixture (50/50%vol.).

Temperature dependences of permeability under an Arrhenius plot are shown in Figure 6 and Figure 7. Calculated apparent activation energies are presented in Table 4.

## 4. Discussion

As can be seen from Table 3, the gas permeability coefficients of the different types of lab-scale membrane modules are similar to each other. Moreover, the results obtained were close to the properties of the same standalone fibers, which had permeability coefficients of 68.4 and 11.9 for CO_2_ and CH_4_, respectively, at 25 °C. These results show that the bores of HFs remained opened. At the same time, the level of CO_2_/CH_4_ selectivity indicates the absence of defects in the HF walls. Both observations allow us to conclude that the developed PMP HFs have sufficient mechanical properties to maintain their membrane characteristics during processing by applied textile methods.

Figure 6 and Figure 7 demonstrate that the temperature dependences of permeability are typical linear dependences under an Arrhenius plot, and fracture near the polymer T_g_ is absent. The CO_2_ and CH_4_ apparent activation energies of permeability in 3D structured HFs were found to be close to the data obtained earlier (Table 4).

**Figure 8:**
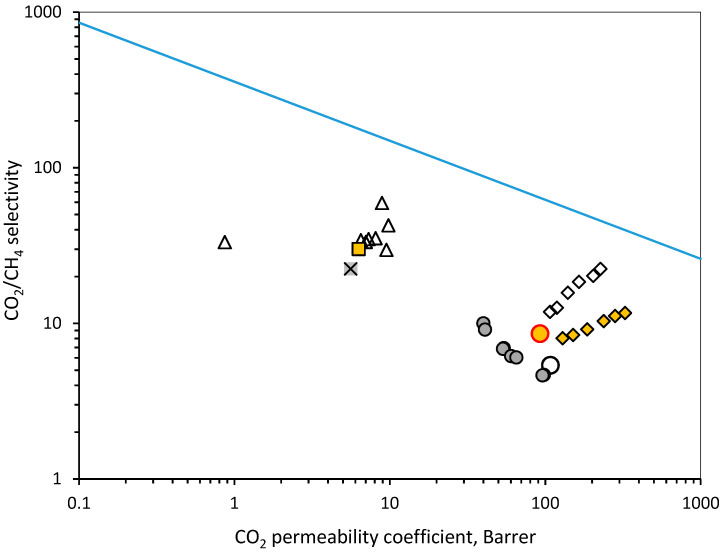
Robeson plot for CO_2_/CH_4_ pair: 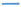 2008 Upperbound; 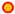 PMP [13]; 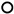 PMP [41]; 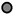 PMP this work (0–40 °C); 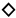 PMP MMMs with MIL53 [42]; 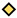 PMP MMMs with Al2O3 [16]; 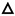 Commercial polyimides [43,44]; 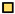 Cellulose acetate [45]; 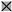 Polysulfone [45].

The comparison of the CO_2_ permeability coefficient and CO_2_/CH_4_ selectivity values obtained in this work with data reported in [41] at 35 °C demonstrates good agreement. However, the value of CO_2_/CH_4_ selectivity reported in [13] is 8.6, which is noticeably higher compared to the value of 5.4 reported in [41] and 5.0 obtained in this work (by interpolation of experimental data for the temperature of 35 °C). Figure 8 shows the comparison as a Robeson plot for the CO_2_/CH_4_ pair, including the values at different temperatures in the studied range 0–40 °C; a decrease in temperature leads to a decrease in CO_2_ permeability in PMP and a rise in CO_2_/CH_4_ selectivity. PMP filling with nanoparticles and the creation of mixed matrix membranes (MMM)s can improve the CO_2_ permeability and CO_2_/CH_4_ selectivity, which was demonstrated in [16,42]. These data added in Figure 8 show that PMP-based MMMs can potentially reach the selectivity level of commercial polymers, which are used for the production of gas separation membranes: polyimides (Matrimid^®®^ and Kapton^®®^) [43,44], cellulose acetate and polysulfone [45]. However, the influence of such PMP modification on the HF melt spinning process has to be studied, and the change in the HFs’ mechanical properties has to be taken into account in order to withstand further braiding or weaving.

Results show that new membrane modules can be obtained from melt-spun PMP HFs using textile processing methods. The most suitable method can be selected depending on the required module geometry and desired hydrodynamic conditions. In the case of HFs with both weaving and braiding, a uniform distribution of fibers without collapse is ensured. The problem of channeling in the shell side, typical of HF membrane modules, can be avoided using modules based on braided structures.

New configurations of membrane modules have the potential to be used not only for typical membrane gas separation systems, but also as gas/liquid membrane contactors for applications in medicine, biology, the creation of protective tissues, contactors and pertractors for various purposes. The development of 3D braided HF modules has the potential to stimulate the creation of membrane systems for new and unusual practical applications. Membranes based on PMP can be applied in gas separation processes, where membranes based on other polymers are not stable. They can be used to separate hydrocarbons [40] or gas mixtures with VOC vapors. Moreover, PMP HFs can be used to create membrane contactors for processes with chemically aggressive absorbents. The potential of HF weaving and braiding using textile techniques is the creation of regular structures that can significantly improve the mass exchange conditions in the shell side of HF membrane modules due to the flow mixing and avoidance of channeling [46].

## 5. Conclusions

The paper presents new approaches for designing HF membrane modules using the form of 3D membrane structures. PMP seems to be practically the only polymer that makes it possible to obtain HFs and 3D braided membrane modules in the form of hoses and fabrics, using the solvent-free “green route”. The proposed textile technique retains the gas transport and separation properties of PMP-based HFs. Taking into account the high chemical resistance of PMP and its stability towards volatile organic compounds, new membrane processes can be developed for separating complex gaseous media (including toxic and reactive components) and creating membrane contactors for application with aggressive absorbents.

## Figures and Tables

**Table 1:** Main trends in improving the efficiency of HF membrane modules.

Purpose of the Membrane Module	Improvement Area	Improvement Method	Remark	Ref.
Gas separation	Increasing the packing density	Modeling (know-how-based technique) on lab-scale HF membrane modules	Recommendations for upscaling gas separation process on industrial membrane modules	[23]
Gas separation	Design of technological systems	Consideration of variable permeability, drop pressure and/or non-isothermal conditions. Multi-component, multi-membrane, multi-operation processes	Possibilities for future applications are discussed	[24]
Hydrogen/alkane separation	Olefins and paraffins C_2_-C_4_ extraction from gas streams	Effects of hollow fiber module design and gas flow mode	Separation of H_2_/CO_2_, H_2_/C_2_H_6_ and H_2_/C_3_H_8_ gas mixtures	[25]
Vacuum sweep for the dehumidification of air	Control of water vapor and air permeance; heat and mass transfer	Varying the geometry of modules and schemes with partial recycling	Decrease in the resistance of the membrane boundary layer towards mass transfer	[26]
Gas/liquid membrane contactors	Mass transfer in HF module	(1) module design with optimized flow geometry (2) use of external energy	Increase in the productivity factor by 3 to 15	[27]
Gas/liquid membrane contactors	Improving mass transfer	Development of a pulsation module that imposes a sinusoidally fluctuating bore liquid flow rate	New method of HF spinning	[28]
HF membrane contactors (HFMC)	HFMC: module fabrication, design and operation, potential applications	Gas/liquid contacting; liquid/liquid contacting; supported liquid membrane; supported gas membrane; fluid/fluid contacting	Special focus on membrane distillation, dehumidification of air and gas absorption and stripping	[29]
Membrane oxygenators	Improvement of hemocompatibility	HF membranes treated by plasma	Surface-modified polymeric HF membranes	[30]
Liquid/liquid membrane contactors	Hydrodynamics and mass transfer	Design of internal HF packing in the module and selection of operating conditions	(1) creating an even flow within the module and (2) improving mixing	[31,32]
Liquid/liquid membrane contactors	HFs with improved homogeneous distribution of fluid inside the module	Dry and wet phase inversion method using asymmetric coagulation conditions	Helix wave HF	[33]
Membrane distillation	Improving the design and mechanical stability of the membrane	Design of lotus-root-like multi-bore HF membrane	Improving the mechanical strength of the membranes	[34]
Membrane distillation	Improving the design of the membrane module	Novel cylindrical cross-flow HF membrane module for direct contact membrane distillation	Good accordance with model predictions	[35]
Power generation from seawater, desalination forward osmosis (FO) and pressure-retarded osmosis (PRO)	Energy efficiency	New types of modules with shorter lengths and larger diameters	Energy recovery increases by 10–15%	[36]
FO	Recuperation factor and energy consumption of FO processes	Experimental and theoretical study of an FO HF membrane module with a cross-wound configuration	Particular attention is paid to the frequency of the transverse winding of the HFs in the module	[37]
Membrane filtration	Fouling	Computational fluid dynamics (CFD) simulations of fiber–fiber interaction in a HF membrane bundle	Fouling can be lowered by proper fiber distance and position in the bundle	[38]
Membrane separation processes	Improvement of mass transfer coefficients	New baffled membrane modules made with HF fabric	The performance of such modules is routinely better than that in more conventional designs	[39]

**Table 2:** Setup and melt spinning parameters of the PMP HF preparation.

Volumetric Polymer Feed (cm^3^/Rotation)	Extruder Rotation Speed (Rotations/min)	Spin Head Temperature (°C)	Specific Mass Throughput (g mm^−2^ h^−1^)	Winding Speed (m/min)
0.16	13.13	280	103	25

**Table 3:** Permeability coefficients of 3D structured HFs for CO_2_/CH_4_ mixture in the temperature range of 0 to 40 °C.

T, °C	Permeability Coefficients *P*, Barrer		Selectivity of CO_2_/CH_4_	Type
	CO_2_	CH_4_		
−0.2	40.0	4.0	10.0	Triaxial braided hose
0.6	41.0	4.5	9.2	Tape-woven fabric
15.2	54.1	7.8	6.9	Triaxial braided hose
15.3	53.5	7.8	6.9	Tape-woven fabric
21.0	60.3	9.8	6.2	Triaxial braided hose
23.5	65.2	10.8	6.0	Tape-woven fabric
39.9	97.9	21.0	4.7	Triaxial braided hose
39.8	95.9	20.7	4.6	Tape-woven fabric

**Table 4:** Apparent activation energies of the gas permeability for different lab-scale membrane modules.

Lab-Scale Membrane HF Module	Apparent Activation Energy of Permeability, *E_P_*, kJ/mol		Reference
	CO_2_	CH_4_	
Industrial “Graviton” HFs	14.3	27.7	[40]
Laboratory HFs	12.9	25.0	[40]
Triaxial braided hose	15.8	29.3	Present work
Tape-woven fabric	15.5	27.7	Present work
